# The pathological role of damaged organelles in renal tubular epithelial cells in the progression of acute kidney injury

**DOI:** 10.1038/s41420-022-01034-0

**Published:** 2022-05-02

**Authors:** Zixian Li, Zejian Liu, Mianna Luo, Xingyu Li, Huixia Chen, Siqiao Gong, Minjie Zhang, Yaozhi Zhang, Huafeng Liu, Xiaoyu Li

**Affiliations:** Institute of Nephrology, and Guangdong Provincial Key Laboratory of Autophagy and Major Chronic Non-communicable Diseases, Affiliated Hospital of Guangdong Medical University, Zhanjiang, 524001 China

**Keywords:** Energy metabolism, Acute kidney injury, Mitophagy

## Abstract

Acute kidney injury (AKI) is a common clinical condition associated with high morbidity and mortality. The pathogenesis of AKI has not been fully elucidated, with a lack of effective treatment. Renal tubular epithelial cells (TECs) play an important role in AKI, and their damage and repair largely determine the progression and prognosis of AKI. In recent decades, it has been found that the mitochondria, endoplasmic reticulum (ER), lysosomes, and other organelles in TECs are damaged to varying degrees in AKI, and that they can influence each other through various signaling mechanisms that affect the recovery of TECs. However, the association between these multifaceted signaling platforms, particularly between mitochondria and lysosomes during AKI remains unclear. This review summarizes the specific pathophysiological mechanisms of the main TECs organelles in the context of AKI, particularly the potential interactions among them, in order to provide insights into possible novel treatment strategies.

## FACTS

- After AKI, mitochondrial dynamics are disrupted, mitophagy and mitochondrial biogenesis disorders are induced. The removal of autophagosomes by lysosomes can be impaired.
- During AKI, the ER produces misfolded proteins, thus, ERS is triggered. The balance between the adaptive and apoptotic pathways of UPR under ERS plays an important role in cell fate.
- Ca^2+^ flux mediated by MAMs and mitochondria-lysosome contact sites are unbalanced in AKI. Screening effective interventions for Ca^2+^ balance in MAMs and mitochondria–lysosome contacts is a potential future research.

## OPEN QUESTIONS

- What are the mechanisms of Ca^2+^ balance protecting the stability of contact among organelles?
- What are other important factors that regulate the stability of contact among organelles?
- How do critical organelles affect each other in different AKI stages?

## Introduction

AKI is a common clinical syndrome, and occurs in approximately 10−15% of the patients admitted to the hospital, while its incidence in intensive care has been reported in more than 50% of patients [1]. Its typical clinical manifestation is a sudden decrease in glomerular filtration rate (GFR), following a rapid increase in serum creatinine levels and/or decrease in urine output, leading to electrolyte disturbance, metabolic acidosis, and volume overload[2]. More seriously, it may be accompanied by dysfunction or failure of other organs [3]. AKI can occur for various reasons, including nephrotoxic drugs, renal ischemia-reperfusion caused by trauma or heart diseases, and sepsis caused by severe infection [4]. The global burden of AKI far exceeds that of breast cancer, heart failure, or diabetes, with remaining high mortality during the past 50 years [5]. Therefore, it is essential to investigate the pathogenesis of AKI and find effective treatments.

TECs are involved in AKI and are also affected by this condition. The proximal tubules, especially the S3 segment, show significant morphological changes in AKI [6]. There are mainly three types of TECs injury outcomes in AKI, i.e., recovery from sublethal injury, apoptosis, and necroptosis. When the injury is not severe, TECs can repair themselves against damage; TECs apoptosis may result from well described extrinsic and intrinsic pathways as well as endoplasmic reticulum stress (ERS), and TECs necroptosis is mainly induced by mitochondrial permeability transition or mediated by prominently receptor-interacting protein kinase-dependent necrotic pathways [7]. In contrast to the heart and brain, the kidney can recover from ischemic reperfusion injury or toxic insult in a timely manner [8]. Depending on a series of cellular biological events, surviving TECs dedifferentiate, proliferate, and migrate to the exposed areas of the renal tubules for tissue repair, which can lead to the restoration of the functional integrity of the nephrons [9].

Cells are the fundamental structural and functional units of organisms, and harbor mitochondria, endoplasmic reticulum (ER), lysosomes, and various other organelles. The orchestrated actions of different organelles maintain cellular homeostasis and survival under pathological stress conditions [10]. However, TECs contain a large number of these organelles, and can be subjected to structural and functional damage due to mitochondrial, ER, lysosomal, and other organelle dysfunction after AKI [11–13]. Interactions among different organelles may aggravate TECs damage or promote the self-repair of damaged TECs, which is one of the key mechanisms affecting the progression of AKI. Here, we summarize the latest studies on the specific pathophysiological mechanisms of the main organelles in TECs during AKI.

## The damage and repair of organelles in TECs during AKI

A variety of organelles may exhibit abnormal function or dyshomeostasis in TECs during AKI. For example, mitochondrial dynamics are disrupted [14], mitophagy and mitochondrial biogenesis disorders are induced [15, 16], and lysosomal homeostasis and quality control are disturbed. The removal of autophagosomes by lysosomes may be impaired [17]. Additionally, during AKI, the ER produces misfolded proteins, and the sterol and lipid metabolism are unbalanced; thus, ERS is triggered [18]. Organelles can interact with each other through multiple signaling pathways, with far-reaching impacts on all aspects of cell and tissue physiology and pathology during AKI [10].

### Mitochondria: The most critical organelle for renal function and most abundant organelle in TECs

Mitochondria are double membrane‒bound organelles found in most eukaryotic cells. They comprise an outer membrane, an inner membrane, an intermembrane space, and a matrix containing its own mitochondrial DNA (mtDNA) [19, 20]. Mitochondria play the crucial function of ATP production through oxidative phosphorylation, which provides a large amount of energy for daily cellular operations. Most of the energy required for cellular life comes from mitochondria, thus, giving it the name “cell power factory” [21]. In addition, mitochondria are involved in many cellular processes, such as differentiation, cell-cell communication, and apoptosis, and have the ability to regulate cell growth and the cell cycle [22].

As an important part of the kidney, renal tubules are functionally quite active, and can reabsorb most of the primary urine, transport glucose, ions, and other substances [23]. Therefore, mitochondria must provide sufficient energy to maintain the normal operation of TECs. However, mitochondria in TECs are vulnerable during AKI, resulting in dysfunction and pathological changes [24]. Mitochondria were severely damaged in the TECs of patients in the early stages of septic shock, showing obvious swelling and flocculent precipitation [25]. Similarly, in animal models in which AKI is induced by renal ischemia-reperfusion or cisplatin, mitochondrial rupture was observed at an early stage, and subsequently apoptotic factors were released to promote apoptosis. Pharmacological inhibition of mitochondrial fission alleviated tubular cell death and renal injury [14].

**Fig. 1:**
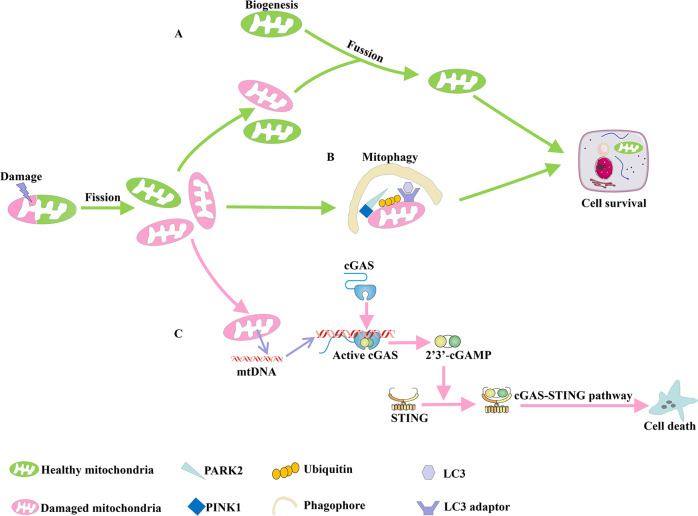
Mitochondrial dynamics during AKI. Mitochondria undergo the dynamic balance of fission and fusion to maintain normal mitochondrial function. **A** In AKI, damaged mitochondria could also occur fission. Then biogenesis and fusion help mix partially damaged mitochondria to produce healthy mitochondria. **B** Additionally, mitophagy are involved in maintaining mitochondrial function and cell homeostasis through degrading damaged or incomplete mitochondria. **C** However, once the dynamics of mitochondria are out of balance, mtDNA is released from damaged mitochondria to the cytoplasm. Subsequently, the cGAS-STING pathway is activated and cell apoptosis occurs. AKI Acute kidney injury, mtDNA Mitochondrial DNA.

Mitochondria undergo constant fission and fusion, biogenesis, and mitophagy to control mitochondrial morphology, quantity, quality, turnover, and inheritance [26]. In contrast, damages to mitochondria not only affect the ATP supply, but also release molecules, such as reactive oxygen species (ROS), cytochrome C, and mtDNA after severe cellular stress, which further induce inflammation, amplify damage, and accelerate cell death [27, 28]. It was shown that in cisplatin-stimulated TECs, mtDNA released by damaged mitochondria leaks into the cytoplasm through Bcl-2 associated protein X (BAX) pores in the mitochondrial outer membrane, with subsequent activation of the cyclic GMP-AMP synthase-stimulator of interferon genes (cGAS-STING) pathway, thereby triggering inflammation and promoting AKI progression (Fig. 1). This was improved in STING-deficient mice [29].

### Disturbance of mitochondrial dynamics in AKI

The mitochondria can undergo continuous fusion and division through complex mechanisms in response to metabolic and environmental stresses, thus maintaining normal mitochondrial function [30]. Fusion helps mitigate stress by mixing partially damaged mitochondria, which can achieve complementation. Fission is needed to create new mitochondria, but it also contributes to quality control by removing damaged mitochondria, and can facilitate apoptosis in the presence of high levels of cellular stress [31]. During AKI, the mitochondrial dynamics of TECs are disturbed, which can result in excessive division and/or suppression of fusion, destruction of the normal morphology of mitochondria, and mitochondrial rupture and dysfunction [14, 32, 33]. Mitochondrial rupture mainly occurs in the proximal tubules and less so in the medullary loop and distal tubules [34].

Mitochondrial fission is dependent on the activation of fission proteins such as dynamin-related protein 1 (DRP1), whereas fusion is controlled in mammals by the outer membrane-anchored dynamin family members, mitofusin gene 1 (MFN1) and mitofusin gene 2 (MFN2), and inner-membrane single dynamin family member, optic atrophy 1 (OPA1) [35–37]. Proximal tubule-specific deletion of DRP1 inhibits mitochondrial fission, reduces oxidative stress, alleviates renal injury, and promotes renal recovery induced by ischemia/reperfusion in TECs in AKI mice [38]. Inhibition of miR-214 could prevent MFN2 downregulation. Compared with wild-type mice, mice with miR-214 knockdown from the kidney proximal tubular cells had less severe tissue damage, fewer apoptotic cells, and better renal function after ischemic AKI. Mitochondrial fusion can inhibit cell death in TECs during AKI [39]. OMA1, a zinc metalloproteinase located in the inner membrane of mitochondria, is responsible for the proteolysis and inactivation of OPA1 during cellular stress [40, 41]. In ischemia-reperfusion-induced AKI mice, OMA1 is activated, and OPA1 is proteolyzed. Compared with wild-type mice, OMA1 knockout mice showed reduced degree of OPA1 protein degradation, mitochondrial rupture, renal tubular injury, and apoptosis under conditions of ischemic stress [42].These findings suggest that changes in mitochondrial dynamics are a typical feature of AKI, and may serve as a potential therapeutic target.

### The controversial protective role of mitophagy in AKI

Mitophagy is the process of selective degradation of defective mitochondria through the autophagy pathway, which is generally recognized as a defense mechanism in a disease state [43]. Emerging evidence has shown that mitophagy plays a protective role in AKI pathogenesis [44].

In ischemia-reperfusion-, cisplatin-, and contrast-induced AKI, mitophagy can be induced to remove damaged mitochondria, prevent the accumulation of ROS, reduce the release of mtDNA and pro-apoptotic factors, reduce inflammation and apoptosis, and ultimately reduce the damage and death of TECs. Conversely, knockout of PTEN-induced kinase 1 (PINK1), parkin RBR E3 ubiquitin protein ligase (PARK2), or BCL2-interacting protein 3 (BNIP3) inhibited mitophagy in TECs, which aggravated kidney injury [45–48]. Under conditions of stress, loss of mitochondrial membrane potential obstructs the entry of PINK1 into the mitochondria, leading to the accumulation of PINK1 on the mitochondrial outer membrane. PINK1 on the mitochondrial outer membrane induces the translocation of PARK2 from the cytoplasm to damaged mitochondria. Ubiquitinated PARK2 is part of the activated form of the mitochondrial outer membrane proteins. Ubiquitin-labeled mitochondria are subsequently recognized by autophagy receptor proteins, such as ATG7, resulting in degradation [49]. Similarly, under hypoxic conditions, BNIP3 acts as a mitophagy receptor that bridges the mitochondria to the autophagosome by directly interacting with LC3 in the autophagosome membrane [50]. In a mouse model of ischemia-reperfusion, mitochondrial loss and autophagosome formation increased in TECs, which could not recover normally [51].

However, Liu et al. showed that activation of mitophagy can aggravate AKI. It has been reported that c-MYC-induced lncRNA MEG3 aggravates kidney ischemia–reperfusion injury by activating mitophagy through the upregulation of RTKN, triggering the Wnt/β-catenin pathway [52].

Generally, it is still controversial whether mitophagy plays a protective role or aggravates disease in the progression of AKI, and more research needs to be conducted. Different AKI models, different mitophagy intervention measures, imperfect mitophagy detection methods, and the interaction of mitophagy and apoptosis pathways are likely the reason for different results [53]. If it is possible to convert the harmful effects of mitophagy into protective factors, one might find better strategies for the treatment of AKI.

### The beneficial role of mitochondria biogenesis in renal repair

To ensure sufficient mitochondria to maintain normal cell operations, mitochondrial biogenesis occurs to replace lost mitochondria [54]. A growing body of evidence supports the beneficial role of mitochondrial biogenesis in renal repair following AKI. The main regulatory factor, PGC-1α (peroxisome proliferator-activated receptor γ coactivator-1α), can directly regulate the expression of a series of transcription factors, such as nuclear respiratory factors and estrogen-related receptors, which then regulate the expression of related nuclear genes in mitochondrial biogenesis [16]. In the kidney, PGC-1α is mainly expressed in the proximal tubules [55]. In renal biopsy samples from patients with AKI, PGC-1α expression is significantly reduced in TECs [56]. In mice with lipopolysaccharide-induced sepsis, the expression of PGC-1α in TECs decreased with the aggravation of renal injury and returned to normal with renal repair. Further studies have shown that systemic or tubule-specific knockout of PGC-1α aggravates kidney damage in septic AKI mice and impedes renal repair [57]. Overexpression of PGC-1α in TECs can alleviate renal injury caused by ischemia-reperfusion AKI and promote renal repair [56]. Similarly, formoterol, a pharmacological stimulant of mitochondrial biogenesis, can accelerate renal function recovery after ischemic reperfusion in mice [58]. These studies suggest that PGC-1α plays a key role in mitochondrial damage and repair by regulating mitochondrial biogenesis in TECs.

### Lysosomes: The central organelle for cellular degradation

Lysosomes are cystic structures encased in a single membrane, which are the main catabolic organelles in cells. They contain a large number of hydrolases, including proteases, nucleases, lipases, sulfates, and phosphatases [59]. Lysosomal degradation involves a wide range of substrates, including a variety of exogenous and endogenous proteins, nucleic acids, polysaccharides, other biological macromolecules, cell organelles, and invading pathogens [60].

In recent years, new discoveries have revealed that lysosomes are not only sites for cellular waste disposal but also highly dynamic structures that mediate intracellular homeostasis to cope with environmental changes [61]. Lysosome dysfunction and depletion can induce blockage of autophagy, which is tightly related to various kidney diseases, including crystalline nephropathy [62], diabetic nephropathy [63], and AKI [64]. Lysosome dysfunction can stagnate the autophagic activity, manifesting as mitochondria dysfunction, the activation of inflammasome and fibrosis in kidney [65]. Thus, better understanding of the mechanisms regulating lysosome function and autophagy pathway may be important for the development of novel therapeutic strategies for kidney diseases.

Furthermore, lysosomes communicate extensively with other cellular structures through physical and functional interactions such as the formation of membrane contact sites [66], vesicles [67] and cytoskeleton [68]. Then it has been reported that the pathological changes of inter-organelles membrane contact sites lead to the development of acute myocardial infarction [69], Charcot-Marie-Tooth disease [70], Parkinson’s disease [71] and kidney diseases [72].

### Lysosomal dysfunction in AKI

In progressive kidney injury, lysosomal functions are partially lost, which prevents the effective degradation of damaged organelles. In mice with drug-induced AKI, oxidative stress, lysosomal damage, and apoptosis of renal cells occur prior to the clinical manifestation of AKI [12]. In mice with cisplatin-induced AKI, zVAD-fmk, a widely used pan-caspase inhibitor, induces lysosomal dysfunction, impedes the cleavage of autophagy proteins, inhibits the autophagic flux, and aggravates renal injury [73]. In cadmium-induced AKI, the epigenetic regulator BRD4 contributes to lysosomal dysfunction, autophagy blockade, and oxidative stress [74].

Cathepsin L(CTSL) is a cysteine proteolytic enzyme of the papain family that is stored in lysosomes as a proenzyme. Under normal conditions, a small quantity of the proenzyme is physiologically secreted into the cytoplasm and can then be hydrolyzed or activated, and is involved in many physiological processes. However, in the pathological state, lysosomal membrane stability decreases, permeability increases, and lysosomes rupture. A large amount of CTSL is released into the cytoplasm or tissue space and is activated to degrade cell or intercellular matrix components [75]. CTSL was shown to play an important role in the initial inflammatory response by activating M1 macrophages in sepsis-induced AKI, which are major contributors to inflammation and tissue damage [76]. In addition, the release of activated CALT from lysosomes into the circulation occurs in conjunction with ongoing local or systemic inflammation, which leads to the development of AKI. Inhibiting CTSL attenuates renal ischemic reperfusion injury by inhibiting inflammasome activation [77].

In the mouse model of tacrolimus-induced renal injury, it was found that LAMP-2A and active cathepsin B levels were decreased and lysosome pH was significantly increased, resulting in lysosome dysfunction and autophagosome accumulation. Klotho treatment can enhance tacrolimus-induced autophagosome clearance by improving lysosomal function [78]. It was found that increased peripheral positioning of lysosomes correlated with increased mTORC activity. mTORC1 activity in renal fibroblasts was increased in a mouse model of lipopolysaccharide-induced AKI [79]. Forcing lysosomes to the periphery prevents them from degrading autophagosomes effectively, causing their accumulation and resulting in an overall reduction in autophagic flux [80].

In addition to the leakage of lysosomal enzymes and abnormal lysosomal intracellular localization, there are also other several mechanisms that can impair lysosome function in AKI, such as elevation of lysosomal pH and lysosomal membrane permeabilization (LMP). The integrity of the lysosomal membrane is essential for lysosomal function. Normally, respiration is the main process used to produce hydrogen peroxide (H_2_O_2_) and ROS in mitochondria [81, 82]. ROS triggers LMP by accumulating iron in lysosomes. In the presence of redox-active free iron, low concentrations of H_2_O_2_ activate a series of Fenton-type reactions that result in the generation of more highly reactive hydroxyl radicals. Hydroxyl radicals attack the lysosomal membrane, destroy its integrity, and lead to its disintegration [83]. The pro-apoptotic protein Bax is also involved in lysosomal membrane integrity regulation by perforating organelles [84]. LMP leads to the leakage of lysosomal contents, resulting in lysosomal dysfunction. In addition, the leakage of lysosomal constituents is thought to be sufficient to trigger cell death in a caspase-dependent or independent manner [85, 86].

During AKI, large amounts of H_2_O_2_ diffuse into lysosomes [83], leading to lysosomal membrane peroxidation and LMP [87]. Subsequently, the content of lysosomes, especially the specific lysosome enzymes are leaked out [77]. Various functions of lysosome are impaired in AKI, i.e., lysosomes are unable to degrade damaged and senescent intracellular organelles and cells, and this further aggravates AKI.

### Autophagic clearance mediated by lysosomes is impaired in AKI

**Fig. 2:**
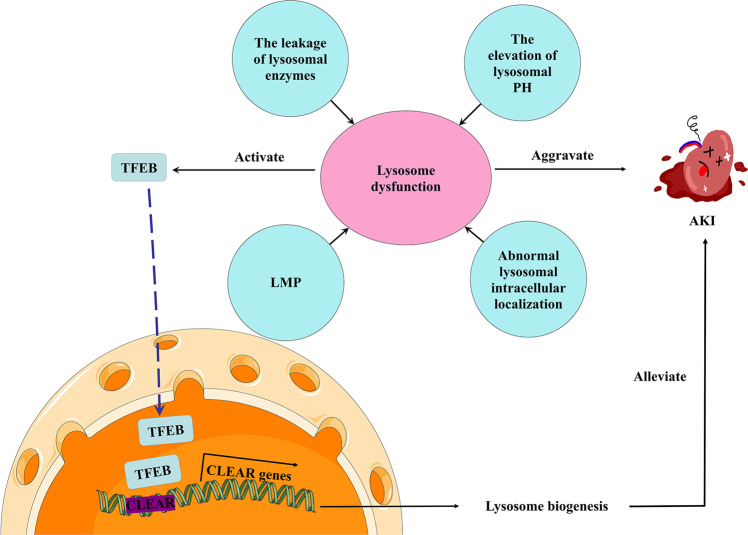
Lysosome dysfunction and TFEB-mediated lysosome biogenesis during AKI. The leakage of lysosomal enzymes, abnormal lysosomal intracellular localization, the elevation of lysosomal pH and LMP could cause lysosome dysfunction, thereby aggravating renal injury during AKI. These pathological conditions induce the nuclear localization of TFEB. TFEB activates CLEAR network genes to control lysosomal biogenesis, which could alleviate AKI. LMP Lysosomal membrane permeabilization, TFEB Transcription factor EB, CLEAR Coordinated lysosomal expression and regulation.

During AKI, lysosomal depletion impairs autophagosome clearance, which aggravates AKI. TECs with autophagosome accumulation are minimally proliferative, with delayed tubular repair after AKI [88]. Disruption of autophagic clearance by the lysosome inhibitor chloroquine aggravates cisplatin nephrotoxicity [89]. Transcription factor EB (TFEB) activates coordinated lysosomal expression and regulation (CLEAR) network genes to control lysosomal biogenesis and autophagy [90, 91]. In mice with ischemia-reperfusion-induced AKI, the expression of activated nuclear-localized TFEB was significantly increased in TECs, and many CLEAR network genes were induced. Ulistone A preconditioning can significantly induce nuclear localization of TFEB and protect the kidney from ischemia-reperfusion injury [92]. Therefore, rescue of lysosome depletion via activation of TFEB-mediated lysosome biogenesis may repair blockage of autophagic flux, suppress apoptosis, and ameliorate AKI (Fig. 2).

### ER: A potentially protective site against AKI progression

ER is a cystic, vesicular, and tubular structure formed by a layer of unit membrane, which forms a continuous omental system. It is called ER because it is near the inner side of the cytoplasm. ER is divided into two types, one is the membrane with ribosome particles, called rough ER; the other kind of membrane is smooth, without ribosomes, called the smooth ER [93]. The smooth ER is mainly involved in the synthesis of sugars and lipids. It can not only supply lipids to itself but also transport synthesized lipids to other organelles, such as lysosomes and mitochondria, to maintain their membranous structure [94].The function of the rough ER is to synthesize proteins and transport them to other compartments within the cell or out of the cell [95]. The function of ER protein-folding machinery is impaired under oxidative stress condition, leading to the accumulation of misfolded proteins in the ER lumen, this condition is referred to as ERS [96]. ERS is induced particularly in AKI caused by ischemic reperfusion injury [18] and nephrotoxicity [97]. Furthermore, ERS is also involved in the pathogenesis of many chronic kidney diseases, including hypertensive nephropathy and diabetic kidney disease [98]. In the kidney, ERS often occurs in tubular cells and podocytes by the stimulation of pathological factors, such as high glucose levels [99] and proteinuria [100]. In order to resist ERS, cells have an integrated signaling system that restores homeostasis and normal ER function. The fundamental pathways that are central to this response include the unfolded protein response (UPR), ER-associated degradation (ERAD), autophagy, hypoxic signaling, and mitochondrial biogenesis [101]. There are three major ER transmembrane proteins, inositol-requiring protein 1 (IRE1), protein kinase RNA-like ER kinase (PERK), and activating transcription factor 6 (ATF6). Under normal physiological conditions, they all bind to the binding immunoglobulin protein (Bip, a surrogate marker of ERS)/78 kDa glucose-regulated protein (GRP78) to form a stable complex [102, 103]. In contrast, under conditions of ischemia/reperfusion [104] or cisplatin-induced [105] AKI, the three ER transmembrane proteins disassociate from Bip, triggering three different pathways to collectively execute the UPR.

### UPR pathways under ERS in AKI

Upon ERS, the UPR is activated, engaging transcriptional, post-transcriptional, and translational programs to reduce the misfolding burden in the ER by reducing the number of proteins entering this compartment. This improves its folding capacity and the clearance of accumulated proteins [106]. The UPR pathway consists of two parts: the adaptive UPR pathway, which maintains ER function or ER protease inhibition, and the apoptotic UPR pathway, which clears dysfunctional cells under severe or long-term ERS [107].

Under various acute pathological stimuli such as hypoxia, the reduction of ATP, nutrient deficiency, and an increase in ROS, the homeostasis of ER proteins is disturbed. The accumulation of misfolded and unfolded proteins will occur. Cells then activate the adaptive UPR pathway, which plays a protective role [108]. For example, in ischemia/reperfusion kidney injury, misfolded and unfolded proteins accumulate in the ER, triggering UPR, which in turn activates IRE-1α and PERK to promote the degradation of unfolded proteins [109]. In addition, ERS-mediated apoptosis can eliminate irreversibly disorganized cells [110].

**Fig. 3:**
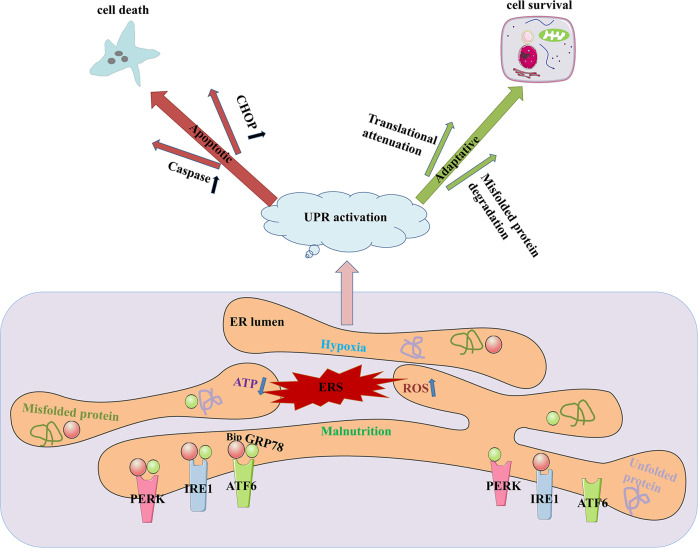
ERS after AKI. Major ER transmembrane proteins mainly include IRE1, PERK and ATF6. Under normal physiological conditions, they are all bound to Bip / GRP78 to form a stable complex. Under various acute pathological stimuli such as hypoxia, the reduction of ATP, nutrient deficiency, and an increase in ROS, tubular cells often undergo ERS. The three ER transmembrane proteins disassociate with Bip to collectively execute the UPR. The UPR pathway consists of the adaptive UPR pathway which could maintain cell function and the apoptotic pathway which could cause cell death. ER endoplasmic reticulum, ERS endoplasmic reticulum stress, IRE1 inositol-requiring protein 1, PERK protein kinase RNA-like ER kinase, ATF6 activating transcription factor 6, UPR unfolded protein response.

However, under severe ERS conditions, when cells are unable to maintain protein homeostasis through the adaptive UPR pathway, the apoptotic UPR pathway is triggered [102]. In a mouse model of ischemia-reperfusion AKI, pharmacological inhibitors of ERS, taurine deoxycholic acid and 4-PBA, attenuated renal tubular cell apoptosis and inflammation and reduced renal tissue damage. In addition, inhibition of ERS after AKI could prevent ischemic kidney injury from developing into chronic kidney disease [111]. Thus, the balance between the adaptive and apoptotic pathways of UPR under ERS plays an important role in cell fate (Fig. 3).

### ERS: A novel therapy target for AKI

In recent years, more and more attention has been paid to the relationship between ERS and AKI. Deng et al. reported that TLR2 inhibitor could suppress ERS-mediated pyroptosis under conditions of sepsis-induced AKI [112]. Human proximal tubular epithelial HK-2 cells express angiotensin (Ang) during renal ischemia, and Ang induces stress granule accumulation and inhibits the translation of ERS-related proteins including C/EBP homologous protein (CHOP, a key marker of ERS) to promote cellular adaptation to ERS. ERS induces more severe renal damage in Ang-knockout mice than in wild-type mice [106].

The bromodomain and extra-terminal domain (BET) protein family consists of four proteins, i.e., Brd2, Brd3, Brd4, and tetris-specific Brdt. Numerous BET protein inhibitors have been shown to regulate cell growth, cell cycle, inflammation, and cancer development [113]. Inhibition of Brd4 with either JQ1 or genetic knockdown resulted in the downregulation of ERS-associated proteins and proapoptotic proteins both in ischemia/reperfusion-induced mouse kidney injury and hypoxia/reoxygenation stimulation in HK-2 cells [18]. Additionally, the expression of CHOP was increased in ischemia/reperfusion kidney injury. Erythropoietin-derived cyclic helix-B surface peptide (CHBP) treatment significantly improved ERS in terms of decreased CHOP expression in kidneys subjected to ischemia/reperfusion injury [109].

According to the above content, it can be seen that ERS is a novel therapy target for AKI. Therefore, we could try to find more effective inhibitors or more targeted pathways of ERS to ameliorate AKI.

## Organelle crosstalk in AKI

Cell compartmentalization allows the segregation and regulation of numerous reactions within the compartments. To maintain the stability of the intracellular environment, cells must transmit signals and exchange materials between compartments and organelles. The dynamically orchestrated actions of all organelles maintain cellular homeostasis [10, 114].

Membrane contact sites (MCSs) are regions in which organelles are closely connected. Intracellular signaling, organelle trafficking, and inheritance occur in MCSs [115]. MCSs, whose functions include the regulation of organelle dynamics, ion and lipid homeostasis, apoptosis, and immune response [116], have received increasing attention and have been associated with a variety of human diseases, such as cancer [117], neurodegeneration [71], kidney diseases [72], and cardiovascular diseases [69].

### Ca^2+^ balance mediated by Mitochondria-associated ER Membranes (MAMs) in AKI

Part of the ER in contact with mitochondria, called the mitochondria-associated ER membranes (MAMs), plays a vital role in Ca^2+^ signaling, lipid homeostasis, mitochondrial dynamics, ERS, apoptosis, inflammation, and autophagy [118]. The stability of MAMs is essential for slowing down the progression of kidney diseases, and the specific relationship between MAMs and AKI requires further study [119].

It has been confirmed that the connection between the ER and mitochondria is composed of protein chains [120]. Based on these highly flexible structural and constitutive characteristics, MAMs can recruit various signaling molecules to maintain intracellular homeostasis [121]. MAMs are essential for transferring Ca^2+^ from the ER to the mitochondria to maintain Ca^2+^ and mitochondrial homeostasis. For example, transient mitochondrial Ca^2+^ uptake by MAMs boosts mitochondrial oxidative respiration and ATP production by stimulating rate-limiting enzymes of the TCA cycle [122] and ATP synthase [123]. Inositol 1,4,5-trisphosphate receptor (IP3R) is a Ca^2+^ release channel located at the ER membrane and has three isoforms, designated as type 1, type 2, and type 3. IP3R2 is the most abundant isoform type in the normal human kidney, particularly in the tubules, while IP3R1 and IP3R3 are expressed at very low levels in the glomeruli and tubules [124]. All three IP3R isoforms have been reported to be involved in MAMs formation. It has been speculated that IP3R2 is the main contributor to the assembly of the Ca^2+^ channel complex in MAMs [125]. Ca^2+^ released from the ER forms Ca^2+^ hotspots at the ER-mitochondrial interface. Ca^2+^ is transported through the mitochondrial intermembrane space and enters the mitochondrial matrix via the mitochondrial Ca^2+^ uniporter [126].

In an adriamycin nephropathy rat model [127], antagonists of the Ca^2+^ regulation axis reduced Ca^2+^ transfer through MAMs and prevented mitochondrial Ca^2+^ overload and subsequent TECs apoptosis, by inhibiting the opening of the mitochondrial permeability transition pore (mPTP) and the release of proapoptotic factors [126, 128]. Furthermore, elevated Ca^2+^ transfer from the ER to mitochondria can result in mtROS overgeneration [72]. However, excessive ROS production may induce oxidative damage to proteins, lipids, and DNA, ultimately leading to renal injury [129]. Importantly, it should be noted that Ca^2+^ uptake by mitochondria not only modifies mitochondrial function, but also alters cytosolic Ca^2+^ activity [127]. Ca^2+^ channel transient receptor potential cation channel subfamily V member 1 (TRPV1) protects against ischemia/reperfusion-induced AKI [130]. In addition, activation of TRPV1 alleviates hyperglycemia-induced mitochondrial dysfunction in podocytes, accompanied by reduced MAMs formation and fewer Ca^2+^ transport from ER to mitochondria [72]. Based on these studies, we speculated that mitochondrial Ca^2+^ disturbance in MAMs is a dangerous signal in mitochondrial dysfunction- and apoptosis-related kidney diseases such as AKI.

**Fig. 4:**
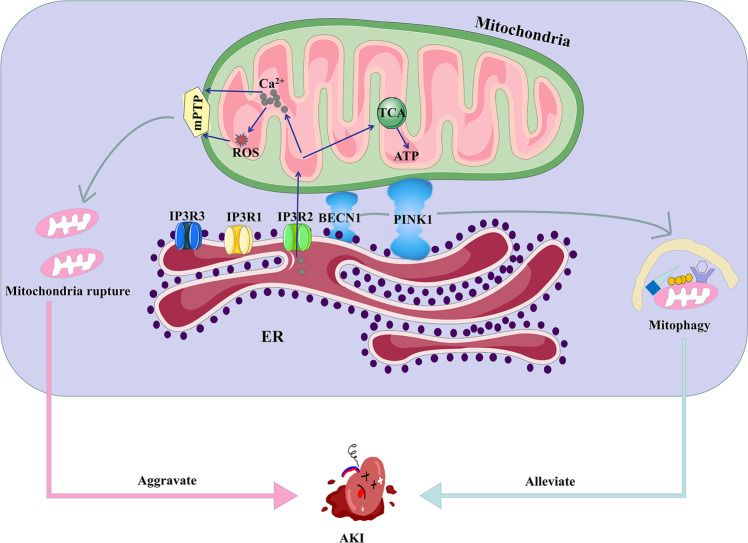
Ca^2+^ balance mediated by MAMs. IP3R is the main Ca^2+^ release channel located at the ER membrane, designated as type 1, type 2, and type 3. Mitochondrial Ca^2+^ overload by MAMs boosts ATP production by stimulating TCA cycles and produces a mass of ROS. The opening of the mPTP results in mitochondria rupture, which could aggravate AKI. However, BECN1/PINK1 expression enhances MAMs formation and promotes mitophagy to alleviate AKI. MAMs mitochondria-associated ER membranes, IP3R Inositol 1,4,5-trisphosphate receptor, mPTP mitochondrial permeability transition pore, BECN1 Recombinant Beclin 1, PINK1 PTEN-induced kinase 1.

Recombinant Beclin 1 (BECN1) and PINK1 are required for autophagosome formation during mitophagy. In a mouse model of Parkinson’s disease, it was found that the direct interaction between PINK1 and BECN1 enhanced the formation of MAMs after mitophagic stimuli [131]. Furthermore, pretreatment with the BECN1/PINK1 peptide induced autophagy and protected against ischemia/reperfusion injury [132, 133]. The clearance of damaged mitochondria via mitophagy is important for the protective effect of ischemic preconditioning in the kidneys [133]. Therefore, manipulation of BECN1/PINK1 expression enhances MAMs formation, promotes mitophagy, and prevents mitochondrial dysfunction and the progression of AKI (Fig. 4).

### Mitochondria-lysosome membrane contact sites

Mitochondrial and lysosomal functions are critical for maintaining cellular homeostasis. Mitochondrial fission is regulated by Drp1, ER, dynamin-2 and actin [36, 134–140]. Damaged mitochondria are targeted to lysosomes for degradation. Lysosomal dynamics are regulated by RAB7 GTPase, which cycles from an active GTP-bound state to an inactive GDP-bound state upon GTP hydrolysis [141]. Active GTP-bound lysosomal RAB7 promotes contact formation. The RAB7 GTPase-activating protein TBC1D15 is recruited to mitochondria by mitochondrial fission 1 (FIS1) to drive RAB7 GTP hydrolysis, thereby mediating contact untethering. The contact sites between mitochondria and lysosomes could provide a potential cellular mechanism for simultaneously regulating these dynamics [66].

Mitochondrial dysfunction and increased oxidative stress have been found in various kidney diseases [27, 142]. Mitochondrial defects and lysosomal dysfunction are common features of renal injury. Renal injury may be partially mediated by defective mitochondria–lysosome contact site function secondary to mitochondrial defects and/or lysosomal dysfunction [66, 143]. Ca^2+^ balance and material exchange via mitochondria-lysosome contact sites are blocked in kidney diseases [144].

**Fig. 5:**
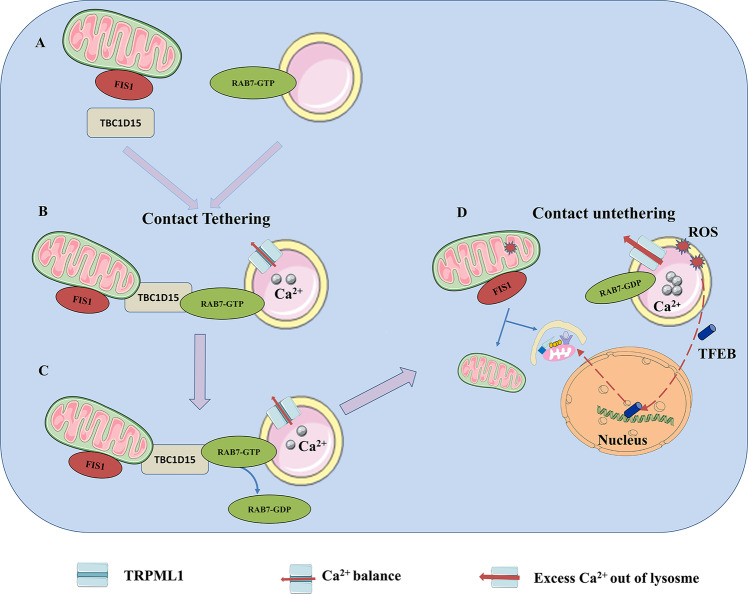
The regulation of mitochondria-lysosome membrane contact. **A** Lysosome and mitochondria could establish crosstalk by mitochondria-lysosome membrane contact. **B** RAB7 GTPase-activating protein TBC1D15 is recruited to mitochondria by FIS1, thereby mediating mitochondria-lysosome membrane contact tethering. **C** Lysosomal dynamics are regulated by the RAB7 GTPase, which cycles from an active GTP-bound state to an inactive GDP-bound state. **D** AKI leads to mitochondria dysfunction, and the mitochondria produce a large amount of ROS. Meanwhile, the lysosomal channel TRPML1, which is the ROS sensor localized on the lysosomal membrane, is triggered, increasing mitochondrial Ca^2+^. The contacts become unstable and untether. In addition, lysosomal damage facilitates Ca^2+^ efflux through TRPML1, which is essential for TFEB activation to induce mitophagy. FIS1 Mitochondrial fission 1, TRPML1 Transient receptor potential mucolipin 1.

It has been found that a function of mitochondria–lysosome contacts is to facilitate the direct transfer of Ca^2+^ from lysosomes to mitochondria. The transfer of Ca^2+^ at mitochondria–lysosome contacts is mediated by the lysosomal channel transient receptor potential mucolipin 1 (TRPML1) [144]. TRPML1, a mucolipin channel on the lysosomal membrane that releases Ca^2+^, functions as a sensor of cellular ROS, which are produced in large part by the mitochondria [145]. In addition, the activity of TRPML1 increases with increasing ROS levels [146]. This activation triggers calcineurin-dependent TFEB nuclear translocation, autophagy induction, and lysosome biogenesis to alleviate renal injury (Fig. 5) [62]. When TRPML1 is genetically inactivated or pharmacologically inhibited, clearance of damaged mitochondria and removal of excess ROS are blocked, aggravating renal injury [147]. A proximal tubule-specific TFEB knockout mouse exhibited progression of oxalate-crystal-induced kidney injury. Oxalate nephropathy is a mouse model of lysosomal damage. Lysosomal damage triggers LC3 recruitment to lysosomes, where lipidated LC3 interacts with TRPML1 to facilitate Ca^2+^ efflux, which is essential for TFEB activation [62]. Therefore, TRPML1 regulates an autophagy-dependent negative feedback program to mitigate oxidative stress.

Generally, AKI leads to mitochondrial dysfunction, and mitochondria produce large amounts of ROS. TRPML1, localized on the lysosomal membrane, senses the production of ROS in large quantities. TRPML1 is triggered and preferentially increases mitochondrial Ca^2+^ at mitochondria–lysosome contacts. TRPML1-mediated lysosomal Ca^2+^ efflux leads to a mitochondrial Ca^2+^ influx [144]. Ca^2+^ homeostasis is beneficial for cleaning damaged mitochondria and removing excessive ROS to improve AKI.

## Conclusion and perspectives

During AKI, critical organelles in the TECs undergo pathological changes. For example, mitochondrial fission and fusion are unbalanced and mitochondrial function is disrupted [14]. Lysosomal membrane integrity is disrupted and leakage of contents results in lysosome depletion [81]. The ER protein-folding machinery interferes with and subsequently leads to the accumulation of misfolded and unfolded proteins in the ER lumen [96].

Understanding how these organelles affect each other is an exciting area of research. MAMs are essential for transferring Ca^2+^ from the ER to mitochondria to maintain Ca^2+^ and mitochondrial homeostasis [125]. Mitochondria-lysosome contacts regulated by RAB7 GTP hydrolysis are unstable in AKI [139]. Activation of lysosomal TRPML1 can maintain Ca^2+^ balance to protect against AKI [144]. Thus, the stability of MAMs and mitochondria-lysosome contacts is critical for the treatment of AKI. Only when MAMs and mitochondria–lysosome contacts are stable can Ca^2+^ transport maintain homeostasis. Ca^2+^ transport homeostasis is beneficial for material transport and energy metabolism [148].

However, the maintenance of the Ca^2+^ balance in unstable MAMs and mitochondria-lysosome contacts after AKI remains a major challenge. During oxidative respiration, the mitochondria continuously pump protons out, resulting in a voltage difference between the inner and outer sides of the mitochondrial membrane. When

Ca^2+^ surrounds the mitochondria, the Ca^2+^ uniporter opens up [149]. Mitochondria then take up Ca^2+^ from the ER by MAMs [150] and Ca^2+^ from the lysosomes by mitochondria–lysosome contacts [144]. In the pathological state of renal toxicity and ischemia, the excessive accumulation of Ca^2+^ in the mitochondria produces ROS, and the electron transport chain of mitochondria continuously produces H_2_O_2_, which increases the resistance of mPTP channel on the mitochondrial membrane. A high resistance state will subsequently obstruct the transportation of Ca^2+^ between MAMs [125] and mitochondria–lysosome contacts [144].

Due to technological limitations, we have not found an effective target or drug to regulate Ca^2+^ balance between MAMs and mitochondria–lysosome contacts after AKI, or even other diseases related to mitochondrial damage. Therefore, Ca^2+^ balance in MAMs and mitochondria–lysosome contacts is a potential future research direction for the treatment of AKI. In addition to Ca^2+^ balance, we also identified other important factors that regulate the stability of contact among organelles.

## Data Availability

Data sharing is not applicable to this article as no datasets were generated or analyzed during the current study.
